# Comment on “Neutrophil-to-Lymphocyte Ratio and Platelet-to-Lymphocyte Ratio: Novel Markers for Diagnosis and Prognosis in Patients with Idiopathic Sudden Sensorineural Hearing Loss”

**DOI:** 10.1155/2015/745879

**Published:** 2015-02-19

**Authors:** Erdim Sertoglu, Huseyin Kayadibi, Metin Uyanik

**Affiliations:** ^1^Biochemistry Laboratory, Ankara Mevki Military Hospital, Anittepe Dispensary, 06580 Ankara, Turkey; ^2^Biochemistry Laboratory, Adana Military Hospital, 01150 Adana, Turkey; ^3^Department of Medical Biochemistry, Gulhane School of Medicine, 06010 Ankara, Turkey

We read with great interest the recently published article by Seo et al. [1] in which the authors evaluated the association of neutrophil-to-lymphocyte ratio (NLR) and platelet-to-lymphocyte ratio (PLR) with idiopathic sudden sensorineural hearing loss (ISSNHL) and its possibility of emerging as a cheap, reliable, and independent prognostic marker of ISSNHL. They found that NLR and PLR values were significantly high in ISSNHL patients. Moreover, they recommended NLR level as a novel potential marker to predict the patients' prognosis in terms of recovery. However, we think that there are some points that should be discussed about the study.

First, NLR, which integrates the detrimental effects of neutrophilia (an indicator of inflammation) and lymphopenia (an indicator of physiologic stress), has emerged as a useful prognostic marker in many studies, as well as studies evaluating patients with idiopathic sudden sensorineural hearing loss [2, 3]. In the original study, despite indicating the presence of any acute inflammation and infection as an exclusion criteria, clinical characteristics provided in Tables 2 and 3 of the original study seem not to be consistent with this information. On the basis of general reference ranges, white blood cell (WBC), neutrophil, and lymphocyte counts of some subjects seem to be compatible with the presence of infection [4]. In such studies aimed at determining predictive markers by using laboratory results, it should be better to identify a specific WBC count range within the exclusion criteria. Determining specific WBC count range as well as clinical conditions likely to affect WBC count could avoid a possible bias in patient selection. Moreover, NLR itself alone without other inflammatory markers (C-reactive protein, erythrocyte sedimentation rate, tumor necrosis factor-alpha, interleukin 6, etc.) has led to the insufficiency of evidence confirming the presence of inflammation and may not accurately provide information about the prognosis of the patients.

Second, although presence of diabetes mellitus (DM) was also specified as another exclusion criterion, as understood from the glucose values presented in Tables 2 and 3 of the original study, some patients with impaired fasting glucose (IFG) and/or impaired glucose tolerance (IGT) were also included in the original study. As is known, NLR values can easily be affected by the existence of inflammation in patients with DM and prediabetes (IFG/IGT) [5]. In a study evaluating the association of NLR with different grades of glucose tolerance and insulin resistance in Asian Indians, it is concluded that NLR can be used as an adjuvant prognostic marker for macro- and microvascular complications in patients with glucose intolerance [6]. Excluding patients with metabolic confounders like IFG and/or IGT, more commonly known as prediabetes, should provide more specific results in the original study.

Third, in the original study, it is noted that binary logistic regression model was used to evaluate these values as independent risk factors for recovery of ISSNHL (Table 4 in the original study). In this model authors evaluated lymphocyte, monocyte, NLR, PLR, and glucose. However, before performing the binary logistic regression analysis, univariate logistic regression analysis and correlation analysis should be performed due to the probable associations among the lymphocyte, NLR, and PLR; then significant parameters derived therefrom should be evaluated by binary logistic regression analysis. As is known, correlation of parameters with each other is very important in binary logistic regression analysis.

In conclusion, we think that the findings of the present study may be affected by confounding factors mentioned above and additional statistical analysis taking all these factors into account should be performed. In such studies aiming at determining a disease-specific cut-off value, being more discerning in patient selection will provide more accurate results. In this way, further information can be obtained about the association between NLR and sudden sensorineural hearing loss.

## References

[1] Seo Y. J., Jeong J. H., Choi J. Y., Moon I. S. (2014). Neutrophil-to-lymphocyte ratio and platelet-to-lymphocyte ratio: novel markers for diagnosis and prognosis in patients with idiopathic sudden sensorineural hearing loss. *Disease Markers*.

[2] Guthrie G. J. K., Charles K. A., Roxburgh C. S. D., Horgan P. G., McMillan D. C., Clarke S. J. (2013). The systemic inflammation-based neutrophil-lymphocyte ratio: experience in patients with cancer. *Critical Reviews in Oncology/Hematology*.

[3] Ulu S., Ulu M. S., Bucak A., Ahsen A., Yucedag F., Aycicek A. (2013). Neutrophil-to-lymphocyte ratio as a new, quick, and reliable indicator for predicting diagnosis and prognosis of idiopathic sudden sensorineural hearing loss. *Otology and Neurotology*.

[4] http://www.mayomedicallaboratories.com/test-catalog/Clinical+and+Interpretive/9109.

[5] Ulu S., Bucak A., Ulu M. S. (2014). Neutrophil-lymphocyte ratio as a new predictive and prognostic factor at the hearing loss of diabetic patients. *European Archives of Oto-Rhino-Laryngology*.

[6] Shiny A., Bibin Y. S., Shanthirani C. S. (2014). Association of neutrophil-lymphocyte ratio with glucose intolerance: an indicator of systemic inflammation in patients with type 2 diabetes. *Diabetes Technology & Therapeutics*.

